# Quality of life in adult patients using dialyzable leukocyte extract for allergic rhinitis

**DOI:** 10.1097/MD.0000000000034186

**Published:** 2023-07-07

**Authors:** Toni Angela Homberg, Ivan Lara, Consuelo Andaluz, Edgar Cervantes-Trujano, Pedro Martín Hernández-Martínez, Sonia Mayra Pérez-Tapia, María Carmen Jiménez-Martínez

**Affiliations:** a Unidad de Servicios Externos e Investigación Clínica (USEIC), Escuela Nacional de Ciencias Biológicas, Instituto Politécnico Nacional, Ciudad de Mexico, Mexico; b Unidad de Desarrollo e Investigación en Bioterapéuticos (UDIBI), Escuela Nacional de Ciencias Biológicas, Instituto Politécnico Nacional, Ciudad de Mexico, Mexico; c Departamento de Inmunología, Escuela Nacional de Ciencias Biológicas, Instituto Politécnico Nacional, Ciudad de Mexico, Mexico; d Laboratorio Nacional para Servicios Especializados de Investigación, Desarrollo e Innovación (I+D+i) para Farmacoquímicos y Biotecnológicos, LANSEIDI-FarBiotec-CONACyT, Ciudad de Mexico, Mexico; e Departamento de Bioquímica, Facultad de Medicina, Universidad Nacional Autónoma de México, Ciudad de Mexico, Mexico; f Departamento de Inmunología, Unidad de Investigación, Instituto de Oftalmología Fundación “Conde de Valenciana”, Ciudad DE Mexico, Mexico.

**Keywords:** allergic rhinitis, dialyzable leukocyte extract, immune modulation, immune regulator, quality of life, rhinoconjunctivitis

## Abstract

Allergic rhinitis (AR) has considerable impact on the general health of individuals. Therefore, treatment trials should include an evaluation of quality of life. We aimed to determine changes in the quality of life of moderate/severe AR patients treated with standard treatment in addition to dialyzable leukocyte extract (DLE), a peptide-based immunomodulator. In a prospective, non-controlled trial, DLE was added to the standard treatment regimen for patients with moderate/severe AR. DLE was administered orally at 2 mg per day for 5 days, followed by 4 mg per week for 5 weeks, and then 2 mg per week for 5 weeks. The primary endpoints were overall improved Standardized Rhinoconjunctivitis Quality of Life Questionnaire (RQLQ) scores, domain scores, and individual item scores of 0.5 points or higher. Statistical significance was defined as *P* < .05. Thirty patients (50% female) aged 14 to 60 years old (33.4 ± 11.9) were enrolled in this study. The mean overall basal quality of life score was 3.41 ± 1.22. After 11 weeks, the mean RQLQ score was 1.74 ± 1.09 (*P* < .0001; 95% confidence interval [CI], 1.05-2.33), and all the domain scores improved (daily activities *P* < .001, 95% CI 0.91–2.15, sleep *P* < .001, 95% CI 0.9–2.26, non-hay fever symptoms *P* = .001, 95% CI 0.51–1.82, practical problems *P* < .001, 95% CI 1.55–2.85, nasal symptoms *P* < .001, 95% CI 1.36–2.67, ocular symptoms *P* < .001, 95% CI 1.05–2.17, emotional *P* < .001, 95% CI 1.23–2.55). Each of the 28 individual item scores on the RQLQ showed clinical (minimal important difference [MID] ≥ 0.5) and statistical (*P* < .05) improvements. DLE might be a beneficial adjuvant treatment for AR. Our results provide preliminary data for future research.

Clinical trials registration ID: NCT02506998

## 1. Introduction

Allergic rhinitis (AR) is a common disease worldwide.^[1]^ The incidence rate in Latin America is approximately 7%^[2]^; in Mexican children, it averages at 15.4% (7.1–28%) with variations, depending on region and degree of urbanization.^[3]^ Disease control in primary care settings is poor and correlates with disease perception.^[4]^

Although not life-threatening, AR considerably affects general well-being, absenteeism, work, and school performance.^[5,6]^ Moreover, its effect on comorbidities such as asthma reflects the need for good treatment options.^[7]^ The impact of AR on sleep, daily activities, and productivity has been assessed in multiple studies, suggesting that quality of life (QoL) evaluation is an important aspect to consider in clinical trials.^[8,9]^

The T-helper cell balance in allergic diseases is skewed toward a Th2/Th17 response, and it has been proposed that Th1/Treg cytokines play a negative regulatory role in Th2 inflammatory predominance.^[10,11]^ Favoring Th1/Treg can thus improve allergic symptoms. Dialyzable leukocyte extract (DLE) is a complex drug^[12]^ made up of a mixture of peptides under 10 kDa in size, whose primary component is ubiquitin.^[13]^ The proposed mechanism of action of DLE is immune modulation, which is initiated by ubiquitin activating its receptor C-X-C chemokine receptor type 4 in the stomach and via the vagus nerve, affecting innate and adaptive signaling pathways and thereby favoring the T-helper (Th1) immune regulatory response.^[14,15]^ With the use of DLE, the production of certain cytokines is clinically modified, including a decrease in tumor necrosis factor alpha and interleukin 6, which drive an immune-regulatory response.^[13–16]^ The administration of DLE improves the clinical response in allergies and other immune-mediated diseases.^[17–19]^

This study aimed to determine changes in QoL of patients with moderate/severe AR, treated with standard treatment in addition to DLE.

## 2. Materials and methods

### 2.1. Subjects

We included 30 patients aged 14 to 60 years with a confirmed diagnosis of moderate/severe AR. The disease was confirmed, and the severity was classified based on the Allergic Rhinitis and its Impact on Asthma guidelines.^[1,20]^ Patients were required to have at least 6 weeks of standard AR treatment with adequately prescribed antihistamines, leukotriene inhibitors, intranasal steroids, intranasal/ophthalmic sodium cromoglycate, and/or immunotherapy in varying combinations before entering the protocol. Confirmation of a type 1 hypersensitivity response through previous positive skin or in vitro allergen testing was required. Asymptomatic patients, those with severe chronic diseases, and those with immunodeficiency during enrollment were excluded. Subjects with incomplete visits were excluded from the analysis. No data substitutions were made.

### 2.2. Study design and treatment

This was a prospective, open-label, non-controlled trial. The standard treatment was continued without any modifications, and DLE (Transferon Oral^®^, National Polytechnic Institute, Mexico) was added. DLE dose and duration of treatment were based on previous recommendations by Berrón-Pérez et al,^[19]^ allowing enough time for a measurable effect to occur as with other allergy medications.^[1]^ The initial DLE dose was 2 mg orally per day for 5 days, followed by 2 mg twice a week for 5 weeks, and then 2 mg per week for 5 weeks. Clinical history was reviewed during each visit (basal, week 1, week 6, and week 11), physical examination was performed, and the Spanish (Mexico) version of the self-administered Standardized Rhinoconjunctivitis Quality of Life Questionnaire (RQLQ) was administered. For patients aged 14 to 17 years, an RQLQ score ≥ 12 was used.

Patients responded to each of the 28 items in the 7-domain questionnaire on a scale of 0 (no affection) to 6 (very bothersome). The total score was calculated from the average score of all items. The scores from each of the 7 domains (activity limitation, sleep problems, nose symptoms, eye symptoms, non-hay fever symptoms, practical problems, and emotional function) were obtained from the average score of the items in each domain. The scores for each item were also obtained. The primary endpoint was a change from baseline in the RQLQ overall scores, domain scores, and individual item scores of at least 0.5, minimal important difference (MID).^[21,22]^ Patients were evaluated by different investigators to avoid systematic error, and double-checking was performed to prevent errors in data transcription.

All patients who underwent DLE signed an informed consent form as part of the IC 13-001 approved by the University Ethics Committee. Patients younger than 18 years also provided oral assent.

### 2.3. Statistical analysis

Statistical analysis was performed using GraphPad Prism software version 6.0f (San Diego, CA) and SPSS version 21 (IBM SPSS Statistics for Windows, Version 21.0, Armonk, NY). The normality of the data was checked for skewness and kurtosis, and was confirmed using the Shapiro–Wilk normality test. Dispersion and central tendency statistics were used to describe variables. A 2-tailed *t* test and one-way analysis of variance were used to determine the differences between the groups and for before and after comparison. Differences were considered statistically significant at *P* < .05, and a ≥0.5-point response on the RQLQ was considered clinically significant. Each item in the RQLQ questionnaire was analyzed.

The sample size was calculated using G*power 3 (Heinrich Heine Universität Duesseldorf) according to the following parameters: Type I error 0.05, power 0.8, and effect size 0.43, based on previous observations.

### 2.4. Patient safety

The presence of any adverse events (AE) associated with the medication was assessed as required by the Mexican Official Standard Norm.^[23]^ Patients whose overall symptoms worsened during the study were considered to have AE. The intention-to-treat population, consisting of all subjects who received at least 1 dose of DLE, was used for safety analysis.

## 3. Results

### 3.1. Subject characteristics

**Figure 1.**
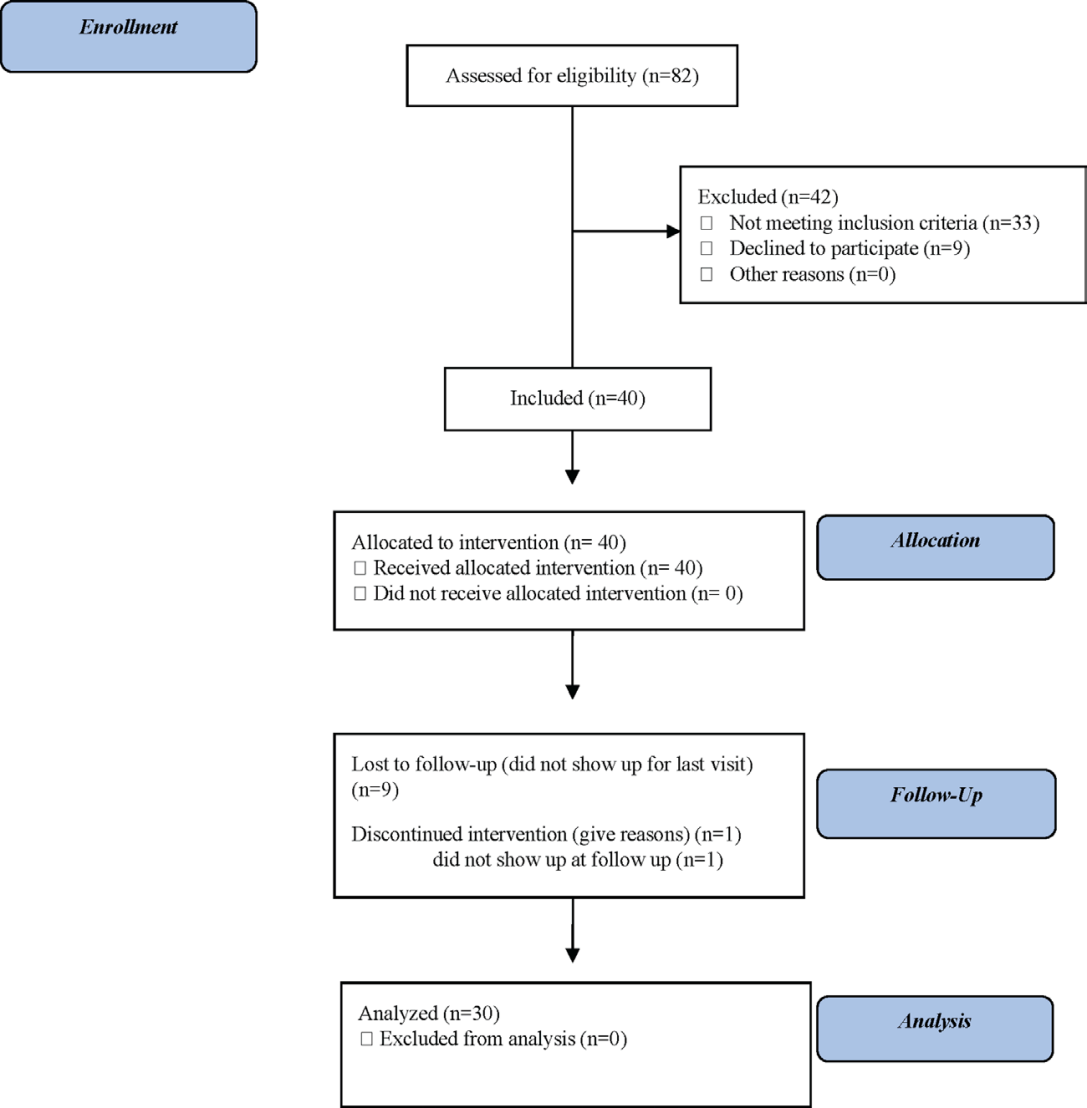
Summary of patient enrollment and study phases.

Thirty patients (50% female) between the ages of 14 and 60 years (33.4 ± 11.9) were followed up for 11 weeks (Fig. 1). Five patients (16.7%) had concomitant asthma, 2 (6.7%) had hypertension, 1 (3.3%) had vitiligo, 1 (3.3%) had atopic dermatitis, 1 (3.3%) had controlled diabetes mellitus, and 1 (3.3%) had controlled hypothyroidism (Table 1).

**Table 1:** Demographic characteristics of the subjects.

	n = 30
Sex	
Female	15 (50)
Male	15 (50)
Age (yr)	
14–17	3 (10)
≥18	27 (90)
# AR medications besides DLE	
1	11 (36.7)
2	15 (50)
3	3 (10)
4	1 (3.3)
Type of AR medications besides DLE	
Antihistamines	13 (43.3)
Nasal steroids	5 (16.7)
Montelukast	5 (16.7)
SLIT/SCIT	2 (6.7)
Sodium cromoglycate (ophthalmic)	1 (3.3)
Comorbidities	
Asthma	5 (16.7)
Hypertension	2 (6.7)
Atopic dermatitis	1 (3.3)
Vitiligo	1 (3.3)
Diabetes	1 (3.3)
Hypothyroidism	1 (3.3)

Values are presented as n (%).

# = number, AR = allergic rhinitis, SCIT = subcutaneous immunotherapy, SLIT = sublingual immunotherapy.

One patient abandoned the study after the first week and was excluded from the efficacy analysis.

### 3.2. Standard treatment

The patients received different standard treatments for AR in various combinations. Twenty-7 patients (90%) were taking antihistamines, 13 (43.3%) were taking nasal corticosteroids, 5 (16.7%) were taking montelukast, 5 (16.7%) were taking sublingual or subcutaneous immunotherapy, and 1 patient (3.3%) had ophthalmic sodium cromoglycate (Table 1). Nineteen patients (63.3%) were taking more than 1 medication for AR. The most common antihistamines used were loratadine and desloratadine in 18 patients (60%). The most common nasal corticosteroids used were fluticasone in three (10%) and budesonide in three (10%).

### 3.3. Overall RQLQ scores

**Figure 2.**
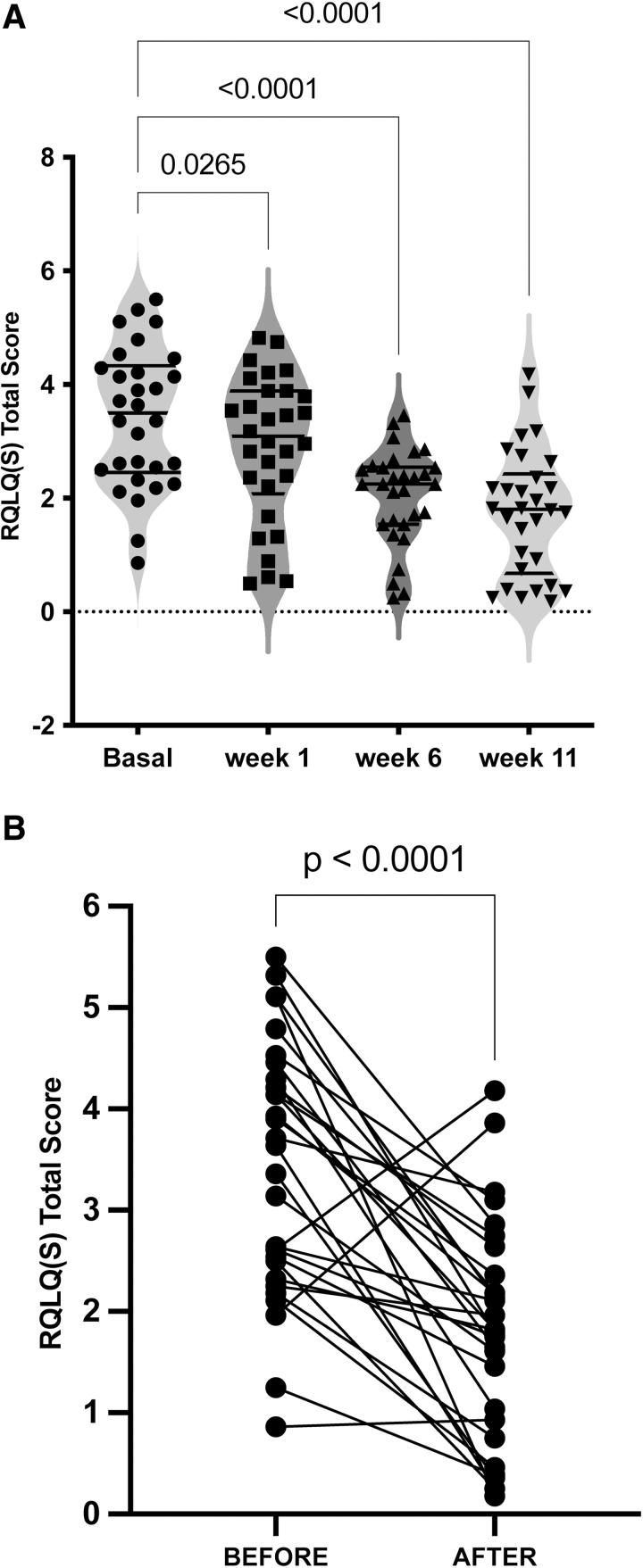
RQLQ(S) score during intervention with DLE. (A) Violin plots showing the distribution of RQLQ(S) total score and the density of subjects expressing the QoL punctuation through DLE intervention. Probability *P* was calculated using a one-way analysis of variance followed by Dunnett’s multiple comparisons test. (B) Before-after RQLQ score analysis showing each patient before intervention with DLE and after 11 weeks of DLE. The probability *P* was calculated using a 2-tailed paired *t* test. DLE = dialyzable leukocyte extract, QoL = quality of life, RQLQ = rhinoconjunctivitis quality of life questionnaire.

The median basal overall RQLQ score before DLE was 3.41 ± 1.22. One week after the addition of DLE, the mean RQLQ overall score was 2.89 ± 1.27 (*P* = .026; 95% confidence interval [CI], 0.05–0.98). After 1 week of DLE use, 14 (46.7%) patients had significantly improved QoL scores (≥0.5). After 6 weeks, the mean RQLQ overall score was 2.05 ± 0.83 (*P* < .0001; 95% CI, 0.83–1.89), and 25 (83.3%) patients had significantly improved QoL scores (≥0.5). After 11 weeks, the mean RQLQ overall score was 1.74 ± 1.09 (*P* < .0001; 95% CI, 1.05–2.33), and 26 (86.7%) patients had significantly improved QoL scores (≥0.5) (Fig. 2; Table S1, Supplemental Digital Content, http://links.lww.com/MD/J220 “RQLQ(S) Total Score” and Table S2, Supplemental Digital Content, http://links.lww.com/MD/J221 “Follow-up RQLQ(S)”).

There was no significant difference in the RQLQ results between sexes, age groups (teens vs adults), or the amount of AR medication taken (Table 2).

**Table 2:** Differences in RQLQ scores between different groups in relation to sex, age group, and number of allergic rhinitis medications before and after 11 weeks of DLE.

Group	n	Mean basal RQLQ	*P*	95% CI	Mean RQLQ after 11 wk	*P*	95% CI
Sex							
Female	15	3.45 ± 1.4	.468	−0.89 to 0.99	1.91 ± 1.2	.336	−0.57 to 1.1
Male	15	3.40 ± 1.10			1.65 ± 1.04		
Age							
14–17	3	2.42 ± 0.27	.041	−2.63 to 0.38	1.23 ± 0.68	.297	−2 to 0.77
≥18	27	3.53 ± 1.25			1.84 ± 1.14		
Number of AR medications							
1	11	3.74 ± 1.16	.53	All intervals contain 0	1.85 ± 1.24	.548	All intervals contain 0
2	15	3.32 ± 1.22			1.9 ± 1.1		
3	3	3.04 ± 1.54			1.27 ± 0.88		
4	1	3.42 ± 1.24			1.78 ± 1.11		

Probability P was calculated using a 2-tailed t test to determine differences in gender and age groups. For differences between groups regarding the number of AR medications, one-way analysis of variance was used.

AR = allergic rhinitis, CI = confidence intervals, DLE = dialyzable leukocyte extract, RQLQ = Standardized version (Spanish-Mexico) of the Rhinoconjunctivitis Quality of Life Questionnaire.

### 3.4. Domain scores of the RQLQ

After 11 weeks of DLE treatment, the mean daily activity score improved from 3.62 ± 1.3 to 2.09 ± 1.25, *P* < .001, 95% CI 0.91–2.15. MID was ≥0.5 in 24 (80%) patients.

The mean sleep symptom score improved from 3.1 ± 1.51 to 1.52 ± 1.33, *P* < .001, 95% CI 0.9–2.26. MID was ≥0.5 in 23 (76.7%) patients.

Clinical improvement was seen in non-hay fever symptoms, and the mean score improved from 2.77 ± 1.67 to 1.6 ± 1.25, *P* = .001, 95% CI 0.51–1.82. MID was ≥0.5 in 19 (63.3%) patients.

Practical problems mean score improved from 4.43 ± 1.46 to 2.23 ± 1.4, *P* < .001, 95% CI 1.55–2.85. MID was ≥0.5 in 25 (83.3%) patients.

The mean nasal symptom score improved from 4.1 ± 1.5 to 2.08 ± 1.24, *P* < .001, 95% CI 1.36–2.67. MID was ≥0.5 in 24 (80%) patients.

The mean score for ocular symptoms improved from 3.2 ± 1.66 to 1.6 ± 1.32, *P* < .001, 95% CI 1.05–2.17. MID was ≥0.5 in 20 (66.7%) patients.

Emotional symptoms improved from 3.48 ± 1.52 to 1.59 ± 1.11, *P* < .001, 95% CI 1.23–2.55. MID was ≥0.5 in 24 (80%) patients.

**Figure 3.**
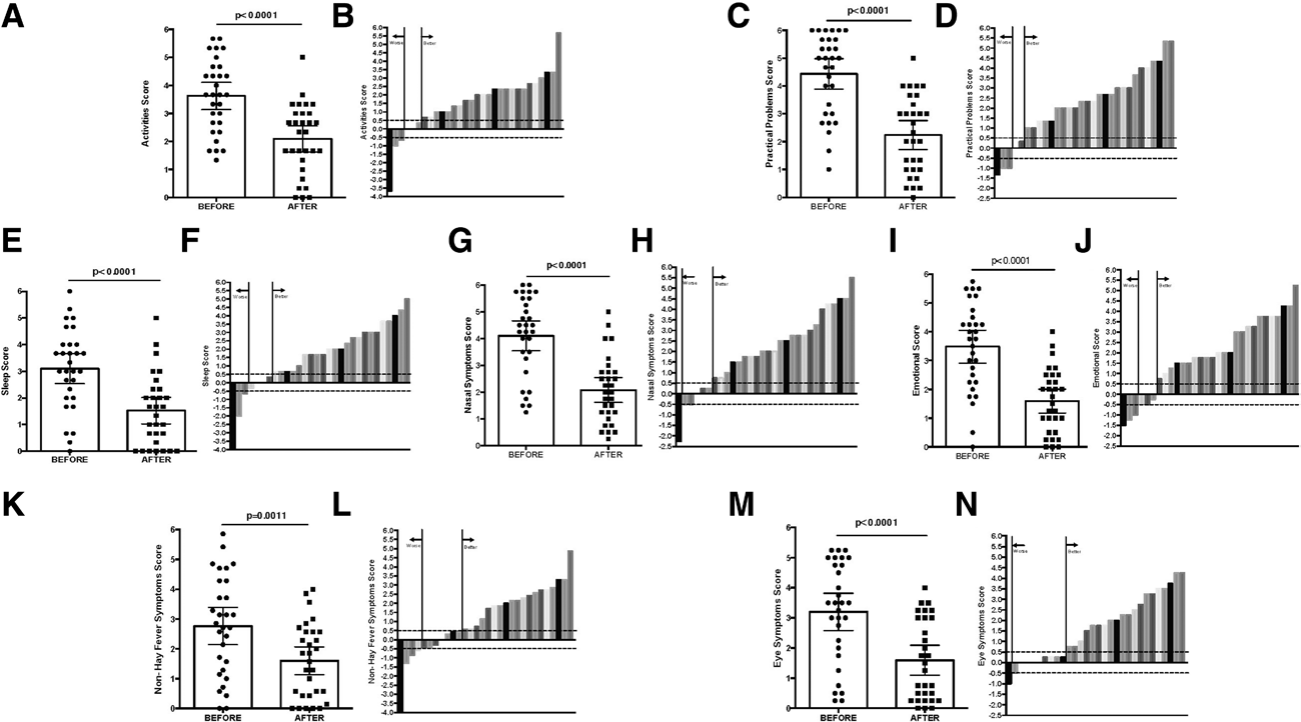
Individual scores per domain in the RQLQ(S) questionnaire. (A, C, E, G, I, K, M) Scores per group of items in the RQLQ(S) before and after adding DLE for 11 weeks, depicting the mean and standard deviation. A 2-tailed paired *t* test was performed to determine the probability, *P*. (B, D, F, H, J, L, N) Magnitude of score improvement per patient for each group of items or symptoms in the RQLQ questionnaire. 0.5 is the MID that relates to significant clinical changes (dotted line). DLE = dialyzable leukocyte extract, MID = minimal important difference, RQLQ = rhinoconjunctivitis quality of life questionnaire.

The scores for each domain before and after 11 weeks of adjuvant DLE are shown in Figure 3. The magnitude of the difference between the basal and final scores per domain for each patient is shown (see also the Table S3, Supplemental Digital Content, http://links.lww.com/MD/J222 “Domain Scores”).

### 3.5. Individual RQLQ Items

Each individual item in the RQLQ questionnaire showed clinical (MID ≥ 0.5) and statistical improvements with the use of DLE (Fig. 3, Column B). The individual items that showed greater improvement in QoL were eye and nose rubbing (86.7%), sneezing (86.7%), nose blowing (83.3%), runny nose (83.3%), social activities (83.33%), and embarrassment (83.3%). The individual symptom with the least improvement was headache (53.3%). Table 3 and Table S4, Supplemental Digital Content, http://links.lww.com/MD/J223 “Items Scores” show the scores for the remaining individual items.

**Table 3:** RQLQ score per item on the questionnaire before and after 11 weeks of DLE.

Item	Basal Score	Final Score	*P*	95% CI	
				Inferior	Superior
Activities at home/work	3.5 ± 1.55	2.17 ± 1.23	.0004	0.645	2.022
Social activities	3.43 ± 1.25	1.97 ± 1.33	<.0001	0.864	2.069
Outdoor activities	3.93 ± 1.6	2.13 ± 1.43	<.0001	1.111	2.489
Difficulty falling asleep	3.2 ± 1.56	1.73 ± 1.34	.0001	0.782	2.151
Waking from sleep	3.1 ± 1.81	1.3 ± 1.39	.0001	0.971	2.629
Not sleeping well	3.0 ± 1.74	1.53 ± 1.43	.0003	0.728	2.205
Fatigue	2.83 ± 1.86	1.77 ± 1.31	.005	0.340	1.793
Thirst	2.9 ± 1.77	1.63 ± 1.3	.001	0.560	1.973
Reduced productivity	2.73 ± 1.86	1.6 ± 1.38	.003	0.415	1.852
Tiredness	3.07 ± 1.91	1.87 ± 1.38	.003	0.444	1.956
Difficulty concentrating	2.97 ± 1.81	1.53 ± 1.41	.0005	0.689	2.178
Headache	2.03 ± 1.96	1.23 ± 1.31	.042	0.032	1.568
Worn out	2.83 ± 1.9	1.57 ± 1.36	.002	0.514	2.019
Inconvenience of needing tissues	4.13 ± 1.87	2.03 ± 1.38	<.0001	1.364	2.836
Rub nose-eyes	4.53 ± 1.43	2.33 ± 1.54	<.0001	1.547	2.853
Blow nose	4.63 ± 1.52	2.33 ± 1.54	<.0001	1.606	2.994
Stuffy nose	4.6 ± 1.59	2.67 ± 1.42	<.0001	1.227	2.640
Runny nose	3.97 ± 1.71	1.8 ± 1.47	<.0001	1.413	2.920
Sneezing	4.07 ± 1.68	2.0 ± 1.44	<.0001	1.388	2.746
Itchy nose	3.77 ± 2.03	1.87 ± 1.46	<.0001	1.126	2.674
Itchy eyes	3.63 ± 2.04	1.97 ± 1.35	<.0001	0.978	2.355
Watery eyes	3.23 ± 1.48	1.6 ± 1.43	<.0001	1.002	2.265
Sore eyes	3.0 ± 1.86	1.4 ± 1.4	<.0001	0.999	2.201
Swollen eyes	2.93 ± 2	1.4 ± 1.48	.0001	0.815	2.252
Frustrated	3.1 ± 1.81	1.63 ± 1.27	.0007	0.672	2.262
Impatient	3.5 ± 1.66	1.63 ± 1.3	<.0001	1.072	2.662
Irritable	3.33 ± 1.9	1.53 ± 1.2	<.0001	1.111	2.489
Embarrassed	4 ± 1.84	1.57 ± 1.25	<.0001	1.735	3.131

Probability *P* was calculated using a 2-tailed t test to determine the differences in scores before and after the addition of DLE.

CI = confidence interval, DLE = dialyzable leukocyte extract, RQLQ = Standardized version (Spanish-Mexico) of the Rhinoconjunctivitis Quality of Life Questionnaire.

### 3.6. Adverse events

None of the patients presented with serious AE. Four patients presented with non-serious AE, 1 patient presented with headache related to the first 2 doses of DLE, and 3 patients showed overall worsened AR symptoms. Table 4 presents the AE in detail.

**Table 4:** Adverse events during the study period.

ID	Age	Male (M)/Female (F)	DLE doses received	Changes on physical examination	# Events per organ system/# exposed	Organ system affected	Severity of adverse event	Causality	Description
49	14	F	20	no	0.10	Respiratory	Low	Probable	Exacerbation of allergic rhinitis symptoms, starting 6 h after the second DLE dose, lasted for 6 d
46	26	M	5	no	0.03	Nervous	Moderate	Probable	Frontal headache 12 h after 2nd and 3rd DLE dose, lasted 4 h, intensity 8/10, took oral ibuprofen 200 mg on 2 occasions, did not recur on subsequent dosing
19	39	M	20	no	0.10	Respiratory	Low	Probable	Increased allergic rhinitis symptoms with nasal congestion and postnasal drip after first DLE dose, did not improve during the 11 wk of DLE treatment
21	51	F	20	no	0.10	Respiratory	Low	Probable	Increased allergic rhinitis symptoms after 11 wk of DLE treatment. Improved at week 15

## 4. Discussion

AR is the most common allergic disease, and treatment options for this persistent disease are limited; therefore, there is a continuous search for new and safe treatment options.^[24]^ To the best of our knowledge, this is the first report of a peptide-based immunomodulator as adjuvant treatment for patients with persistent moderate/severe AR. Previous clinical observations at our research unit suggested that DLE improved AR and asthma symptoms,^[25]^ but no similar studies have measured QoL in patients using DLE. This study is an important step in quantifying clinical improvement by administering the RQLQ questionnaire before and after each treatment phase, including dosing changes.

Owing to its mechanism of action that diminishes the Th2-inflammatory response,^[14]^ DLE might have similar effects to specific immunotherapy; thus, it is expected to be used concomitantly with other medications during exacerbations. Nonetheless, the long-term goal of specific immunotherapy is to decrease the need for additional medications and to reduce the severity of allergic symptoms. Although this is the first step in the study of DLE as an adjuvant for AR, the long-term goal is to decrease the year-long dependency of patients on topical steroids, antihistamines, and leukotriene antagonists.

The total RQLQ scores in our study showed clinically significant improvements, surpassing the minimal required 0.5-point change in score, aside from showing statistical significance after 11 weeks of DLE treatment. The QoL improvement observed in this study was well above the required MID and exceeded that of other immunomodulators, such as specific immunotherapy and probiotics. A real-world clinical study of sublingual and subcutaneous immunotherapy that used RQLQ as a primary endpoint reported MID after 6 months of subcutaneous immunotherapy.^[26]^ Another study used data from four Phase III trials of grass pollen and tree pollen sublingual immunotherapy tablets to determine new MIDs per allergen and season, suggesting that the same MID cannot be applied to all scenarios because it might vary by allergen, pollen exposure at the time of the study, treatment, and other factors. However, all the new MIDs were below the usually required 0.5 points.^[27]^ Another study that evaluated the effect of lactobacilli versus placebo on RQLQ scores showed no clinically significant improvement, although the results were statistically significant.^[28]^ This suggests that DLE may have similar or improved immunomodulatory properties. Nonetheless, a more modest effect is expected in real-life situations.

As each patient was compared with her/himself, the type of standard treatment was irrelevant even if it remained unchanged. Despite the differences in the number of AR medications taken, we found no difference in QoL improvement between patients taking one, two, three, or four different AR medications. According to the guidelines, patients with persistent symptoms of AR require one or two medications (nasal steroids with or without oral antihistamines), not including immunotherapy. A third medication (leukotriene antagonist) may benefit pediatric patients with AR, and some may occasionally use local vasoconstrictors.^[1]^ A study of children and adolescents with different AR phenotypes showed that 62% to 67% of patients with persistent AR remained symptomatic regardless of the guideline-recommended treatment,^[29]^ causing some prescribers to overstep the treatment guidelines to help their patients. An increased number of medications increases treatment costs,^[30]^ and DLE seems to improve QoL with no relation to the number of other AR medications taken.

All 7 domains (daily activity limitation, sleep problems, nose symptoms, eye symptoms, non-hay fever symptoms, practical problems, and emotional function) in the RQLQ questionnaire showed statistical and clinical improvements during DLE treatment. Each of the 28 individual items in the questionnaire also showed statistical and clinical improvement. Bousquet et al^[31]^ found that ocular symptoms had the highest impact on QoL, followed by nasal obstruction and pruritus, to a lesser degree.

The main symptoms of AR are sneezing, nasal congestion, pruritus, and nasal secretion.^[3]^ The items in the questionnaire with the most improvement were sneezing, nasal congestion, pruritus (rubbing of nose/eyes, itchy nose) and nasal secretion (nose blowing, inconvenience of carrying tissues, runny nose). This suggests that DLE can be beneficial for improving the main symptoms of AR. As expected, due to the psychometric qualities of the questionnaire, the individual item score improvement was consistent with the domain score and overall RQLQ score improvement.^[32]^ This allows us to conclude that there was a consistent and general improvement in the QoL of AR patients who had DLE added to their treatment.

Despite the DLE dose reduction in each phase, we observed a significant improvement in the RQLQ scores over time, suggesting that DLE dosing once a week may be adequate for most patients. However, we cannot exclude the possibility that a higher initial dose is beneficial. Additional studies comparing the efficacies of different doses are required to resolve this issue.

The most common AE associated with the use of DLE are headache, increased disease symptoms, rash, and fatigue, which are present in less than 2% of DLE users.^[33]^ Three patients (10%) in this study presented with non-serious AE that correlated with the type of adverse event reported by Homberg et al^[33]^ Interestingly, the symptom that least improved was headaches, which is consistent with the finding that DLE may cause headaches.

The limitations of this study include the small number of subjects, lack of a control group, and the fact that only QoL was evaluated. Due to the sample size and lack of a control group, no definite conclusions can be made regarding efficacy of DLE until larger, controlled, multicenter studies involving different populations are done. Nonetheless, this study provides encouraging results in favor of continuing the study of DLE as a possible treatment option for AR. QoL is one of the many tools available for assessing patients with AR.^[9,32]^ It is helpful to guide treatment options and responses; however, the scores are based on the patient’s subjective point of view and personality traits. Nevertheless, DLE appears to be beneficial regardless of basal treatment. Additional studies are necessary to evaluate subgroups of patients with similar traits. Evaluation of symptom scores, visual analog scales, and Th1/Th2/Th17/Treg cytokine profiles is required to fully determine the effectiveness of DLE treatment for AR.

## 5. Conclusions

DLE may be beneficial as adjuvant treatment for AR. Our results provide preliminary data for future research. Interestingly, the patients showed additional improvement with increased treatment time, and dose reduction did not have a worsening effect.

## Acknowledgments

The authors wish to thank Elizabeth Juniper and her team for allowing us to use the RQLQ questionnaire.

## Author contributions

**Conceptualization:** Toni Angela Homberg, María Carmen Jiménez-Martínez.

**Data curation:** Toni Angela Homberg, Ivan Lara, Consuelo Andaluz.

**Formal analysis:** Pedro Martín Hernández-Martínez, Toni Angela Homberg, María C. JIménez-Martínez.

**Funding acquisition:** Sonia Mayra Pérez-Tapia.

**Investigation:** Toni Angela Homberg, Ivan Lara, Consuelo Andaluz, Edgar Cervantes-Trujano.

**Methodology:** Ivan Lara, Consuelo Andaluz, Edgar Cervantes-Trujano, Pedro Martín Hernández-Martínez, María Carmen Jiménez-Martínez.

**Project administration:** Toni Angela Homberg, María Carmen Jiménez-Martínez.

**Supervision:** Sonia Mayra Pérez-Tapia, María Carmen Jiménez-Martínez.

**Validation:** Pedro Martín Hernández-Martínez, María Carmen Jiménez-Martínez.

**Visualization:** María Carmen Jiménez-Martínez.

**Writing – original draft:** Toni Angela Homberg.

**Writing – review & editing:** Sonia Mayra Pérez-Tapia, María Carmen Jiménez-Martínez.

## Supplementary Material








